# Supplementary material for: STRling: a k-mer counting approach that detects short tandem repeat expansions at known and novel loci
Source: Genome Biol. 2022 Dec 14;23:257. doi: 10.1186/s13059-022-02826-4 (PMC9753380; doi:10.1186/s13059-022-02826-4)
Supplement: Supplementary file 1 — Additional file 1: Table S1. Novel STR diseases. Table S2. Sensitivity of ExpansionHunter denovo run on PCR-free Illumina WGS of 94 subjects with alleles of pathogenic size at an STR disease locus. EHdn was run in outlier mode once for each subject against 260 individuals from the 1000 genomes project. Table S3. STRling false discovery rate when compared to long reads. STRs were called using STRling on ~30x Illumina WGS of the Ashkenazim trio. STRling calls >20 bp were verified by if there was a matching insertion in the PacBio HiFi assembly from the same sample (see Methods). Figure S1. Simulated allele size at the HTT locus predicts the number of anchored and unplaced pairs. Figure S2. STRling sensitivity (A) and false discovery rate (B) as a function of allele size. Figure S3. STRling underestimates allele sizes in Fragile X Syndrome (FXS). Figure S4. STRling locus discovery and outlier detection by sample size. Figure S5. STRling resource usage. Figure S6. ExpansionHunter Denovo resource usage. Supplementary Methods. [file 13059_2022_2826_MOESM1_ESM.pdf]

Additional file 1: Supplementary Tables, Figures, and Methods

Table S1: Novel STR diseases

Disease	Gene	Repeat unit	Genic position	Reference
Bartela-Scott syndrome	*XYLT1*	CCG	noncoding	LaCroix 2019
BAFME/FAME1, 6 and 7	*SAMD12* et al	TTTTA + TTTCA	intronic	Ishiura 2018 Nature Genetics
SCA37		TTTTA + TTTCA	intronic	Seixas et al 2017 AJHG
Glutaminease deficiency		CAG	5'UTR	van Kuilenburg 2019 NEJM
Neuronal Intranuclear Inclusion Disease		GGC	5'UTR	Tian 2019 AJHG, Sone 2019 Nat Genet, Ishiura 2019 Nat Genet
FAME2		TTTTA + TTTCA	intronic	Corbett (unpublished)
FAME3		TTTTA + TTTCA	intronic	Florian (unpublished)
CANVAS	*RFC1*	AAGGG<br>ACAGG	intronic	Cortese 2019<br>Scriba 2020

Table S2: Sensitivity of ExpansionHunter denovo run on PCR-free Illumina WGS of 94 subjects with alleles of pathogenic size at an STR disease locus. EHdn was run in outlier mode once for each subject against 260 individuals from the 1000 genomes project.

AD: Autosomal Dominant, AR: Autosomal Recessive, XD: X-linked Dominant, XR: X-linked Recessive. Novel STR disease loci (not in reference genome) are indicated in **<u>bold/underline</u>**. Repeat units are reported on the forward strand.

Disease	Inheritance	repeat unit	CG%	Significant outlier	N subjects
**<u>CANVAS</u>**	AR	AAGGG	60	5 (100%)	5
**<u>DBQD2</u>**	AR	CCG	100	1 (100%)	1
DM1	AD	CAG	66.7	18 (100%)	18
DM2	AD	CCTG	75	1 (100%)	1
DRPLA	AD	CAG	66.7	2 (100%)	2
FRDA	AR	AAG	33.3	26 (100%)	26
FTDALS1	AD	GGGGCC	100	1 (100%)	1
FXS	XD	CGG	100	16 (100%)	16
HD	AD	CAG	66.7	13 (100%)	13
SBMA	XR	CAG	66.7	3 (100%)	3
SCA1	AD	CTG	66.7	3 (75.0%)	4
SCA3	AD	CTG	66.7	2 (100%)	2
SCA6	AD	CAG	66.7	0	1
SCA8	AD	CTG	66.7	1 (100%)	1
**Total**				**92 (96.8.%)**	**95**

Table S3: STRling false discovery rate when compared to long reads. STRs were called using STRling on ~30x Illumina WGS of the Ashkenazim trio. STRling calls >20 bp were verified by if there was a matching insertion in the PacBio HiFi assembly from the same sample (see Methods).

		all loci	2-6bp	significant outliers	significant outliers 2-6bp
hg002	FDR	0.48	0.16	0.50	0.16
	TP	326	87	168	58
	FP	301	17	168	11
hg003	FDR	0.45	0.17	0.47	0.17
	TP	365	86	194	59
	FP	295	17	171	12
hg004	FDR	0.47	0.18	0.47	0.16
	TP	339	79	183	54
	FP	300	17	164	10
Aggregate	FDR	0.47	0.17	0.48	0.16
	TP	1030	252	545	171
	FP	896	51	503	33

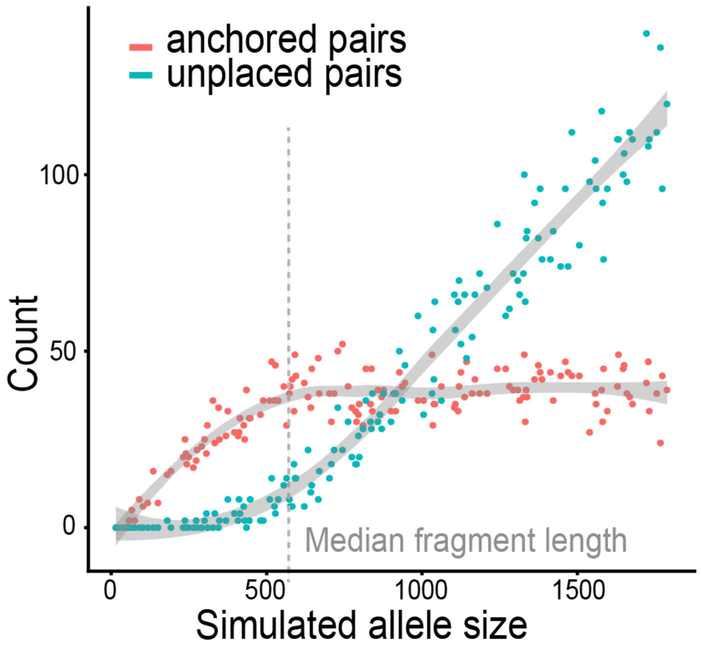

Figure S1: Simulated allele size at the HTT locus predicts the number of anchored and unplaced pairs. The number of anchored pairs is approximately linearly correlated with allele size until the fragment length, while the number of unplaced pairs is approximately linearly correlated with allele size beyond the fragment length.

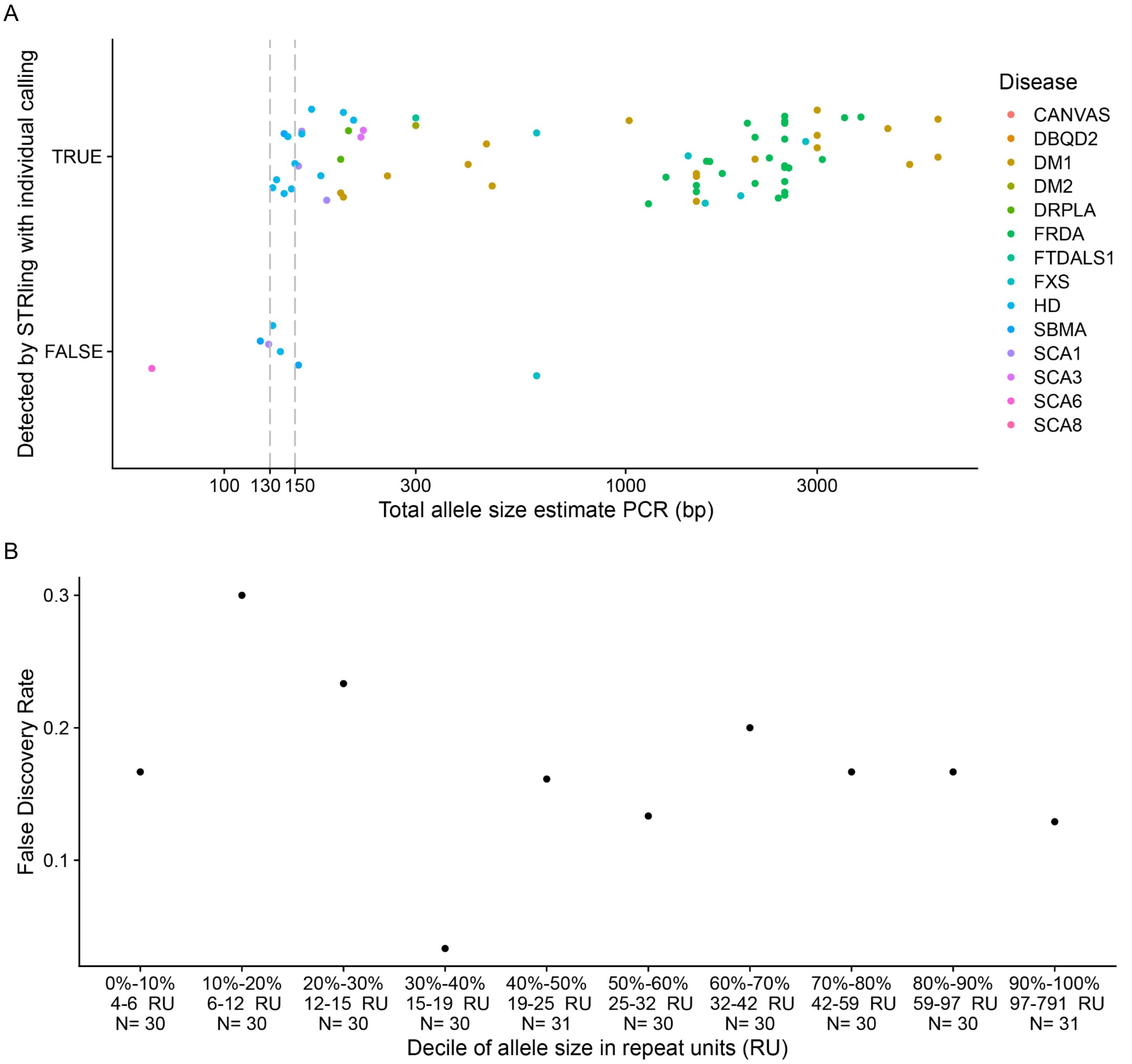

Figure S2: STRling sensitivity (A) and false discovery rate (B) as a function of allele size. A: Orthoginally validated true positive pathogenic STR expansions plotted by total allele size in bp reported by PCR and categorized by whether STRling detected the expansion with individual calling. STRling missed alleles smaller than 130bp, and was most likely to detect alleles larger than 150bp. Most of the calls in this size range are HD. **B:** The set of STRling 2-6bp repeat unit variants assessed for False Discovery Rate (FDR) using PacBio (Supplementary Table 3) were divided into ten equally sized groups (deciles) based on STRling predicted allele size. We report the FDR for each decile.

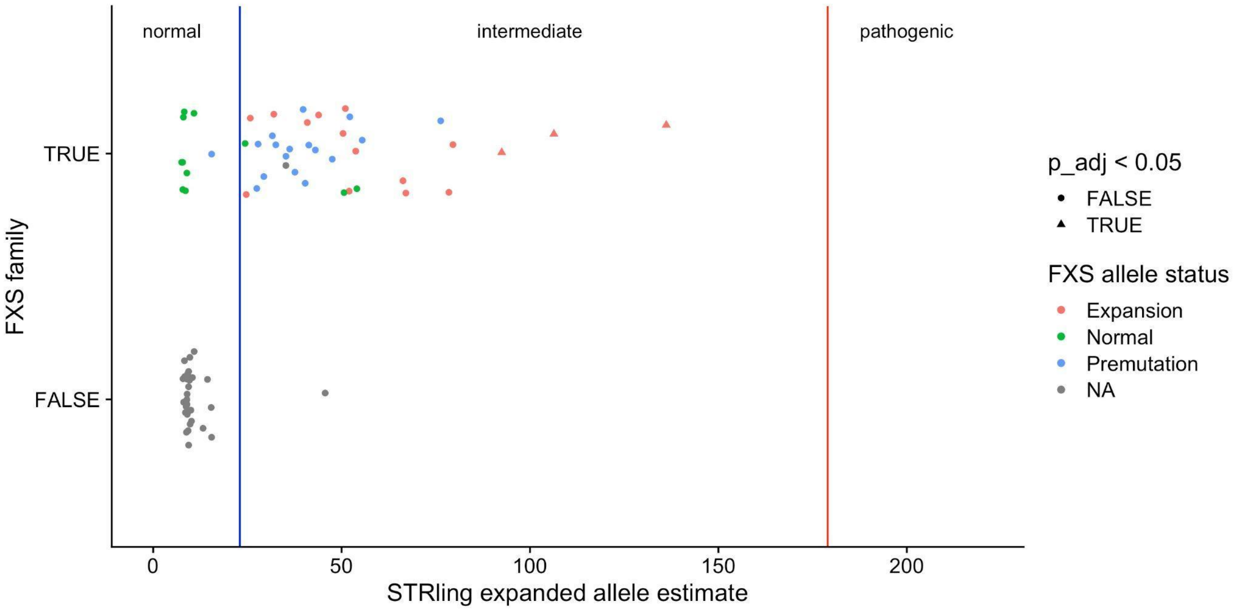

Figure S3: STRling underestimates allele sizes in Fragile X Syndrome (FXS). STRling was run on a cohort of X individuals, including X individuals who were tested for the FXS expansion by PCR. Individuals designated Expansion had a FXS allele with at least 200 CGG repeats. Premutation was 45-200 repeats, normal below 45. The remaining samples indicated NA were not tested, but are assumed to have no pathogenic FXS expansion. Individuals who were outliers at the FXS locus compared to controls are indicated by p_adj < 0.05.

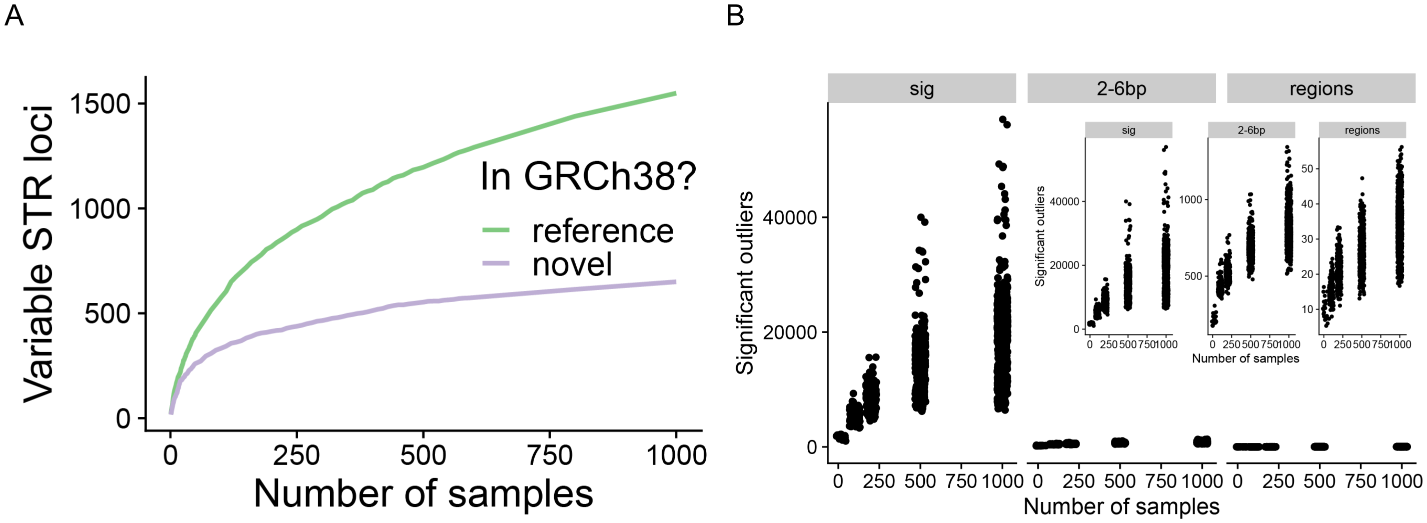

Figure S4: STRling locus discovery and outlier detection by sample size. A: STRling joint calling was performed on 1000 individuals from the 1000 Genomes Project then the resulting calls were subsampled to smaller sets of individuals. A locus was considered variable if the allele size was at least 50 bp and it was supported by at least 5 soft-clipped reads in at least one individual. B: We randomly sampled individuals from the 1000 Genomes Project, performed STRling joint calling on each subset, and reported the number of significant outliers per individual. All outliers on canonical chromosomes chr1-22, X, and Y, outliers at 2-6bp repeat unit loci, and outliers at 2-6bp repeat unit loci excluding those overlapping LCRs, segmental duplications, centromeres, or telomeres. The inset shows the same data but with differing y axes.

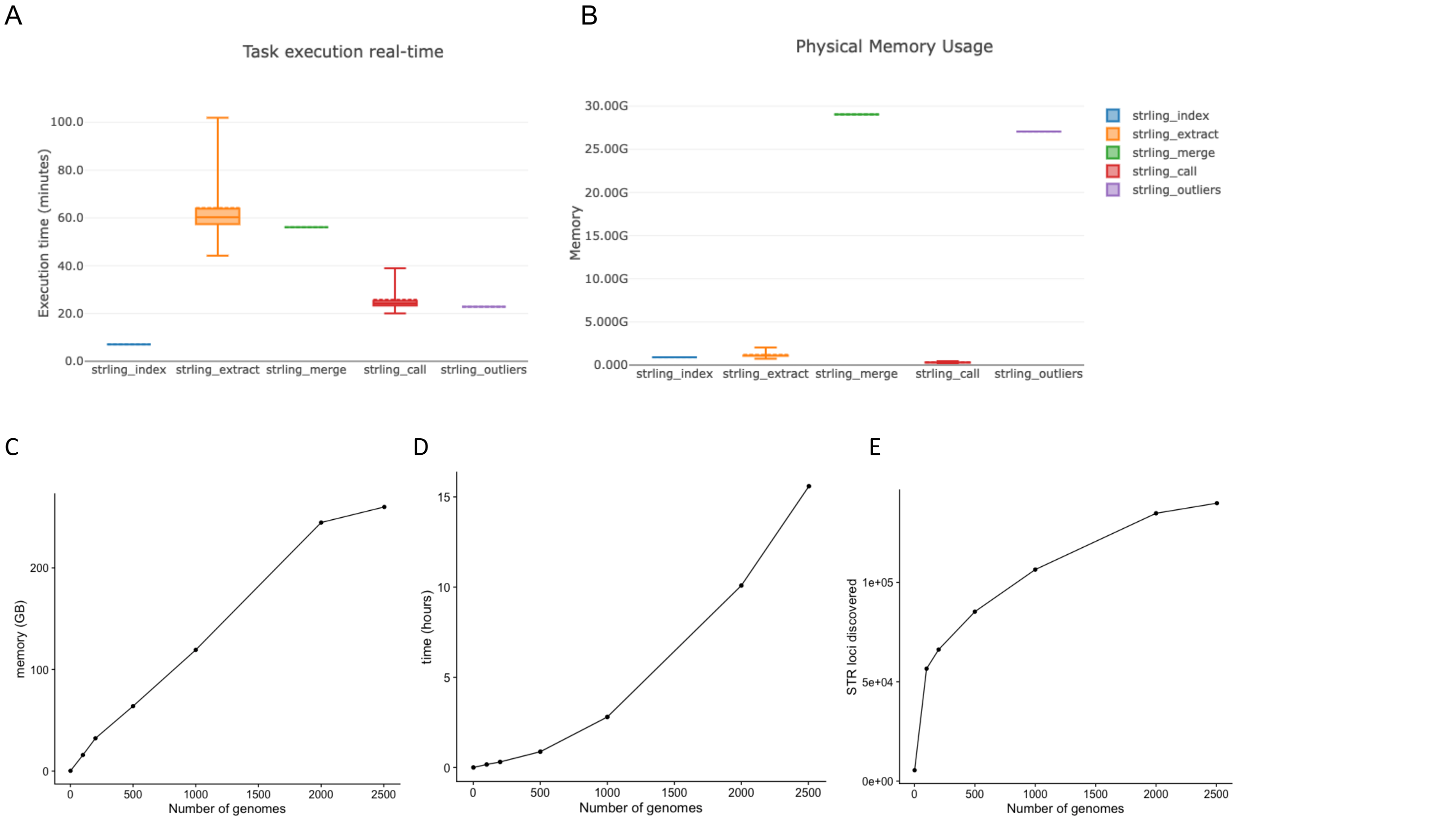

Figure S5: STRling resource usage. A STRling joint-calling Nextflow workflow was executed on 260 WGS from the 1000 Genomes Project. **A**: Time and **B**: memory usage. **C-D**: In these scenarios, the STRling "merge" stage was applied to subsets of only 1, 100, 200, 500, 1000, 2000 and the full 2504 genomes from the 1000 Genomes Project. Samples are first randomly ordered, then select the first N samples, such that each sample contains all samples from each smaller one.

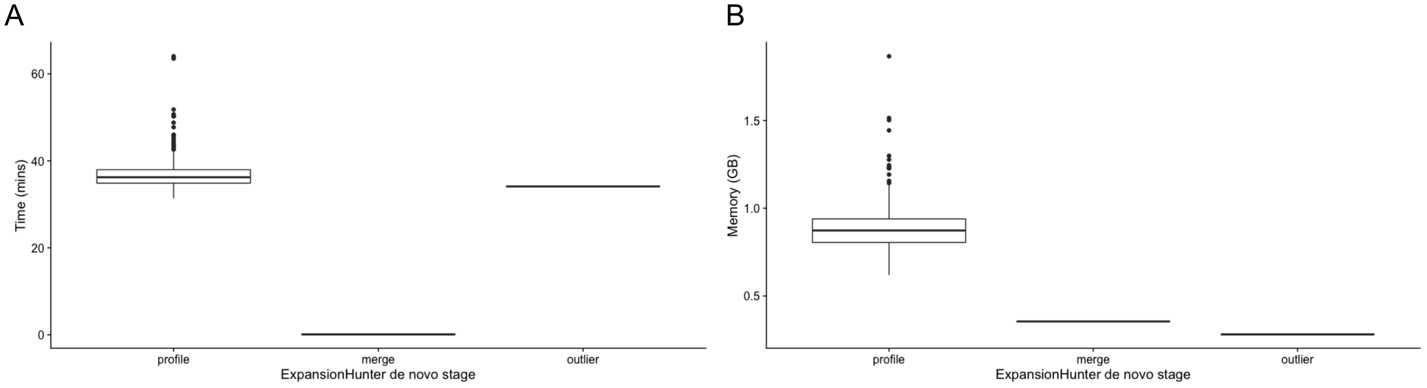

Figure S6: ExpansionHunter Denovo resource usage. A EHdn outlier workflow was executed on the same 260 WGS from the 1000 Genomes Project. **A**: Time and **B**: memory usage.

Supplementary Methods

PacBio assemblies

Sequencing reads are available on SRA. HG002: SRR10382244, SRR10382245, SRR10382248 and SRR10382249. HG003: SRR11567494 - SRR11568082. HG004: SRR11568075 - SRR11568077, and SRR11568083 - SRR11568088.

The PacBio Improved Phased Assembler (IPA) was run on default for all assemblies using the following commands and versions:

Command:

```
ipa dist -i reads.fofn --nthreads 24 --njobs 30 --cluster-args 'qsub -S /bin/bash -N ipa.{rule}
-cwd -q bigmem -pe smp {params.num_threads} -e qsub_log/ -o qsub_log/ -V' --tmp-dir $TMP
```

Software versions:

ipa.py ipa (wrapper) version=1.1.2

snakemake version=5.17.0

ipa2-task 0.5.0

Machine name: 'Linux'

falconc version=1.8.0+git.63e589a80f5668e1cfe0a0ac0f26e2f51501a1ca, nim-version=1.3.5

Nighthawk 0.1.0 (commit 28d8475)

pancake 0.2.0 (commit 881d3bc)

pblayout 0.1.0 (commit 64f78e5)

racon version=1.4.13-cb13104

samtools 1.10

Using htslib 1.10.2
